# Stringency of the 2-His–1-Asp Active-Site Motif in Prolyl 4-Hydroxylase

**DOI:** 10.1371/journal.pone.0007635

**Published:** 2009-11-05

**Authors:** Kelly L. Gorres, Khian Hong Pua, Ronald T. Raines

**Affiliations:** 1 Department of Biochemistry, University of Wisconsin–Madison, Madison, Wisconsin, United States of America; 2 Department of Chemistry, University of Wisconsin–Madison, Madison, Wisconsin, United States of America; The Scripps Research Institute, United States of America

## Abstract

The non-heme iron(II) dioxygenase family of enzymes contain a common 2-His–1-carboxylate iron-binding motif. These enzymes catalyze a wide variety of oxidative reactions, such as the hydroxylation of aliphatic C–H bonds. Prolyl 4-hydroxylase (P4H) is an α-ketoglutarate-dependent iron(II) dioxygenase that catalyzes the post-translational hydroxylation of proline residues in protocollagen strands, stabilizing the ensuing triple helix. Human P4H residues His412, Asp414, and His483 have been identified as an iron-coordinating 2-His–1-carboxylate motif. Enzymes that catalyze oxidative halogenation do so by a mechanism similar to that of P4H. These halogenases retain the active-site histidine residues, but the carboxylate ligand is replaced with a halide ion. We replaced Asp414 of P4H with alanine (to mimic the active site of a halogenase) and with glycine. These substitutions do not, however, convert P4H into a halogenase. Moreover, the hydroxylase activity of D414A P4H cannot be rescued with small molecules. In addition, rearranging the two His and one Asp residues in the active site eliminates hydroxylase activity. Our results demonstrate a high stringency for the iron-binding residues in the P4H active site. We conclude that P4H, which catalyzes an especially demanding chemical transformation, is recalcitrant to change.

## Introduction

**Figure 1:**
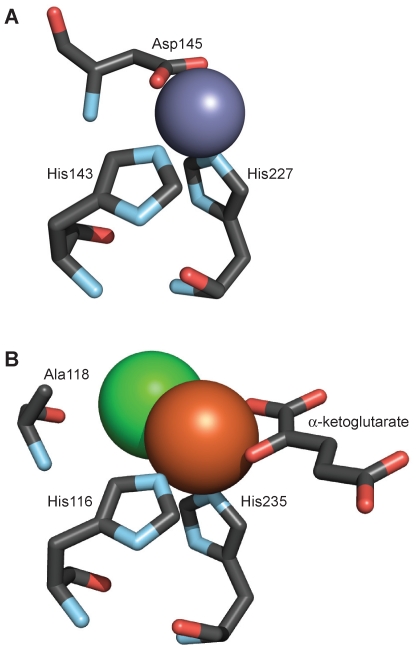
Active sites of α-ketoglutarate-dependent iron(II) dioxygenases. (A) The *Chlamydomonas reinhardtii* prolyl 4-hydroxylase contains a 2-His–1-carboxylate facial triad composed of His143, His227, and Asp145. A zinc ion (gray) replaced iron in the crystal structure [28]. (B) The SyrB2 halogenase has two histidine residues (His116, His235) that coordinate to the iron ion (orange). α-Ketoglutarate is also bound to the iron. Alanine (Ala118) is found in place of the carboxylate-containing residue of hydroxylases, allowing space for the chloride ion (green) [12].

Iron is a common cofactor in enzymes that employ molecular oxygen as an oxidant. In addition to those iron-dependent enzymes that rely on heme, there is a class of non-heme, mononuclear iron(II) enzymes that catalyze the hydroxylation of aliphatic C–H bonds, dihydroxylation of arene double bonds, epoxidation of C–C double bonds, heterocyclic ring formation, and oxidative aromatic ring opening. The iron in these non-heme dioxygenases is commonly coordinated by a 2-His–1-carboxylate motif [1]. Two histidine (His) and either one aspartic acid (Asp) or one glutamic acid (Glu) residue bind the iron at the vertices of one triangular face of the octahedral metal center, forming a 2-His–1-carboxylate triad of facial ligands (Figure 1A). This arrangement leaves three coordination sites on the iron open for the substrate, oxygen, and other co-substrates.

A subset of non-heme iron(II) enzymes uses α-ketoglutarate as a co-substrate. These dioxygenases also contain the 2-His–1-carboxylate iron-binding motif. The substrate binds near the active site, while α-ketoglutarate binds directly to the iron in a bidentate manner. The reaction mechanism involves oxidative decarboxylation of α-ketoglutarate to produce succinate, CO_2_, and a high energy Fe(IV)-oxo intermediate [2]. In a hydroxylation reaction, one atom of oxygen is incorporated into the product, and the other into succinate.

**Figure 2:**
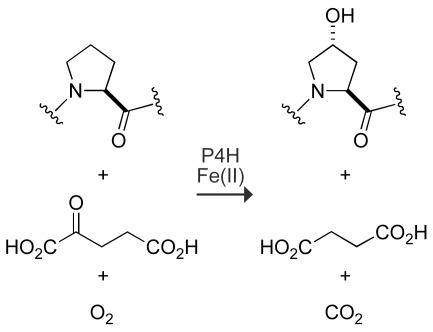
Reaction catalyzed by P4H.

Prolyl 4-hydroxylase (P4H) is an α-ketoglutarate-dependent, iron(II) dioxygenase, and catalyzes the post-translational hydroxylation of proline residues (Pro) during collagen biosynthesis [3]. This hydroxylation reaction forms (2*S*,4*R*)-4-hydroxyproline (Hyp; Figure 2), which is necessary for the proper folding of the collagen triple helix [4]. Accordingly, P4H is an essential enzyme for animals [5]–[7].

The active site of P4H is within the α subunit of the α_2_β_2_ enzyme tetramer. The β subunits (protein disulfide isomerase) keep the α subunits soluble and retained within the endoplasmic reticulum [8], [9]. The 2-His–1-carboxylate iron-binding residues of P4Hα, identified by site-directed mutagenesis, comprise His412, Asp414, and His483 [10], [11].

A recently discovered subclass of α-ketoglutarate-dependent, iron(II) dioxygenases catalyzes halogenation reactions in natural product biosynthesis. The halogenase SyrB2, found in *Pseudomonas syringae*, catalyzes the conversion of threonine to 4-chlorothreonine during the biosynthesis of syringomycin E. The three-dimensional structure of SyrB2 revealed a chloride ion present in place of a carboxylate ligand in the facial triad [12]. The Asp or Glu present in other mononuclear iron enzymes is replaced by alanine (Ala) in SyrB2, creating space in the active site for the binding of a chloride ligand to the iron (Figure 1B). The halogenase CytC3 likewise contains a 2-His motif rather than the 2-His–1-carboxylate facial triad [13].

**Figure 3:**
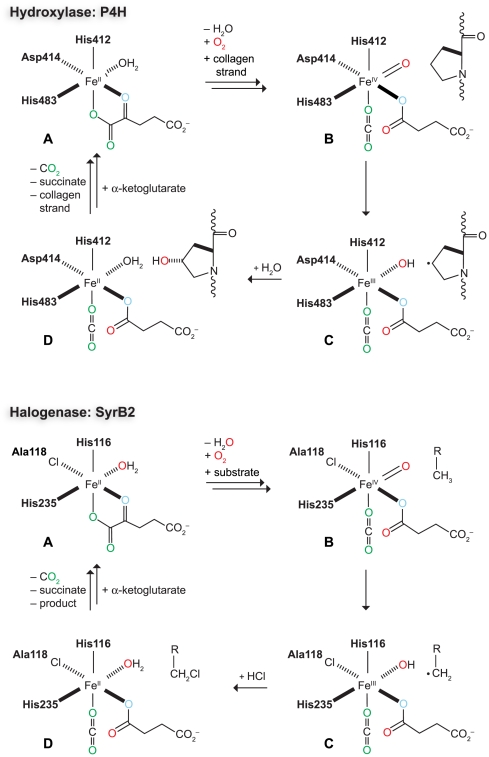
Proposed reaction mechanisms for the hydroxylase P4H (top) and halogenase SyrB2 (bottom). In both mechanisms, the intermediates are labeled **A**–**D**. **A** Iron(II) in the active site is bound by a 2-His–1-Asp motif in P4H, but a 2-His–1-chloride in SyrB2. The configuration of these residues is not known for human P4H, but is drawn in analogy to SyrB2 (Figure 2B). **B** The reactive iron(IV)-oxo species is formed upon decarboxylation of α-ketoglutarate. **C** The iron(IV)-oxo species abstracts a hydrogen atom from the substrate producing a radical intermediate. **D** In the hydroxylase reaction, the substrate radical recombines with the hydroxyl group. In the halogenase reaction, the substrate radical reacts with the chloride ligand.

Despite the difference in iron-binding residues, halogenation is thought to follow a mechanism similar to that of P4H and other α-ketoglutarate-dependent iron(II) dioxygenases (Figure 3). Decarboxylation of α-ketoglutarate generates a reactive Fe(IV)-oxo intermediate, which abstracts a hydrogen atom from a C–H bond of the substrate [14], [15]. This reaction yields a substrate radical and a Fe(III)-OH complex. Hydroxylated products result from the recombination of the substrate radical with the iron-coordinated hydroxyl group. In contrast, the radical substrate intermediate of halogenases combines with the chlorine atom coordinated to the iron, instead of the hydroxyl group, to produce a chlorinated product. This discovery prompts the question of how the enzyme controls product formation.

Herein, we test the stringency of the iron-binding ligands in P4H. We determine whether altering the P4H active site so as to mimic that of a halogenase can convert its hydroxylase activity into halogenase activity. In addition, we characterize the spatial relationships within the active site of P4H by changing the relative positions of the residues that constitute the 2-His–1-carboxylate facial triad. The results provide insight on enzymes that catalyze difficult but important chemical transformations.

## Results

### Difference between Hydroxylases and Halogenases

**Figure 4:**
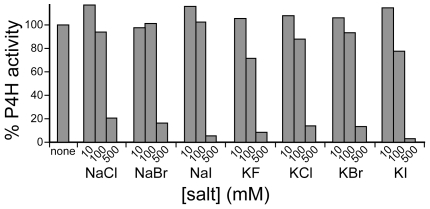
Tolerance of P4H activity to salts. P4H activity is shown in the absence and presence of increasing concentrations of sodium chloride (NaCl), sodium bromide (NaBr), sodium iodide (NaI), potassium fluoride (KF), potassium chloride (KCl), potassium bromide (KBr), and potassium iodide (KI). Reaction mixtures are described in the Materials and Methods section.

P4H, an α-ketoglutarate-dependent iron(II) dioxygenase, contains a 2-His–1-carboxylate motif. In related enzymes that catalyze halogenation reactions, instead of hydroxylations, the carboxylate-containing residue is replaced with Ala, providing space for a halide ion to bind to the iron. If the only difference between hydroxylation and halogenation is an aspartate versus an alanine, then exchanging these residues should interconvert the activities. The halogenation reaction requires the presence of halide ions. Accordingly, we first investigated the activity of P4H in the presence of salts. Up to 100 mM KF, NaCl, NaBr, NaI, KF, KCl, KBr, or KI had little effect on P4H activity (Figure 4). Higher salt concentrations (500 mM) decreased P4H activity by ∼80%.

The D414A variant of P4H, which has an active site analogous to that of SyrB2, was produced, and, as expected [16], lost its hydroxylase activity. Still, no halogenated product from reaction mixtures containing D414A P4H was detected by high-performance liquid chromatography (HPLC) or mass spectrometry, even in the presence of 100 mM sodium fluoride, chloride, or bromide (Table 1). Subsequently, a D414G variant was made to provide even more space for a halide ion within the active site. This variant likewise had no detectable halogenase or hydroxylase activity.

**Table 1:** Biochemical attributes of P4H variants.

P4H variant	Poly(proline) affinity	Hydroxylase activity	Halogenase activity
Wild-type	+	+	−
D414A	+	−	−
D414G	+	−	−
D414H	+	−	nd
D414H/H412D	+	−	nd
D414H/H438D	+	−	nd

nd, Not determined.

### Small Molecules Cannot Replace the Carboxylate in the P4H Active Site

Functional groups missing from amino-acid side chains can be restored by small molecules that rescue protein function [17]. In the D414A and D414G variants of P4H, the carboxylate group is absent. The sodium salt of azide, formate, or acetate (100 mM) was added to D414A P4H reaction mixtures, but did not lead to the formation of hydroxylated product. Nor was the activity of D414G rescued by nitrite or nitrate.

### A 3-His Active Site Is Not Functional in P4H

A 3-His active site is found in cysteine dioxygenase, a non-heme, iron(II) dioxygenase related to P4H that does not utilize α-ketoglutarate. To mimic that enzyme, Asp414 was replaced with a histidine residue. The D414H variant was, however, unable to form a hydroxylated product detectable by either the HPLC-based enzyme assay or mass spectrometry.

### Spatial Orientation of the 2-His–1-Asp Triad Is Critical for Activity

The 2-His–1-Asp triad occupies adjacent coordination sites on the iron. The relative position of these residues varies with respect to the sites occupied by α-ketoglutarate and oxygen [18]. The locations of the two histidine and aspartate residues were shuffled in the H412D/D414H and D414H/H483D variants. No hydroxylated product was detected in reactions with either of these P4H variants by either HPLC or mass spectrometry. Apparently, the relative positions of the components of the 2-His–1-carboxylate triad are critical for the hydroxylation activity of P4H.

## Discussion

The 2-His–1-carboxylate motif is common to many α-ketoglutarate-dependent iron(II) dioxygenases. The related halogenases differ in that the iron is coordinated by only two His residues. In the halogenases, Ala is located in place of the Asp/Glu, and a chloride ion binds to the iron in place of the carboxylate ligand. In an attempt to coerce P4H to perform halogenation reactions, we created the D414A and D414G variants of P4H. Although these variants lose their hydroxylase activity, they do not gain detectable halogenase activity. Similar experiments have been conducted in other dioxygenases to explore the conversion of hydroxylases to halogenases, and vice versa. Replacing alanine with aspartate or glutamate in the halogenase SyrB2 abolished halogenase activity with no observable hydroxylase activity [12]. Substitution of aspartate for alanine in hydroxylase taurine dioxygenase (TauD) abolished hydroxylase activity with no gain in halogenase activity [19]. The D101A variant of TauD did bind iron, though not as efficiently as did the wild-type enzyme, but it was not known if sufficient chloride ion was binding in the active site. The affinity of D414A P4H and D414G P4H for iron was not determined, as even the wild-type P4H does not bind iron with high affinity (*K*_M_ = 5 µM [20]). These P4H variants were able to be produced like the wild-type enzyme and had a similar affinity for poly(proline) (Table 1), as assessed by their retention during poly(proline)-affinity chromatography. Accordingly, we conclude that global protein conformation was unaffected by the amino-acid substitutions.

All data thus far shows that the difference between Fe(II) and α-ketoglutarate-dependent hydroxylases and halogenases extends beyond the presence or absence of an enzymic carboxyl group. Analysis of structural data of the halogenases CytC3 and SyrB2 suggests that residues that do not make a direct contact with the active-site iron are essential for binding α-ketoglutarate and a chloride ion [12], [13]. Residues that surround the active site form a network of hydrogen bonds, which appear to contribute to chloride binding in CytC3 and SyrB2, and could fail to do so in the P4H variants.

The hydroxylase activity could not be rescued by a number of small molecules. Chemical rescue of the residues within the 2-His–1-carboxylate motif of α-ketoglutarate-dependent iron(II) dioxygenases has been demonstrated with other enzymes. The activity of the H174A variant of phytanoyl-CoA 2-hydroxylase was rescued with the addition of imidazole [21]. Interestingly, the same substitution at the other iron-binding His residue was inert to rescue. Deleterious substitutions of the carboxylate ligand have also been rescued by small molecules, as demonstrated by the rescue of TauD D101A by formate [19]. These data provide clues about the accessibility of the enzymic active sites, as well as the tolerance to deviation in ligand position.

Although the 2-His–1-carboxylate motif is common to many iron(II) α-ketoglutarate-dependent dioxygenases, there are variations of the metal coordination motif [22]. Whereas the 2-His–1-carboxylate dioxygenases are quite selective for iron, enzymes containing three histidines and one glutamate (3-His–1-Glu) coordinate additional metals. Moreover, some dioxygenases do not utilize a carboxylate ligand at all. The halogenases, discussed previously, contribute just two histidine residues for iron binding. Enzymes with four histidine (4-His) and three histidine (3-His) ligands have also been identified. A comparison of the 3-His and 2-His–1-carboxylate metal sites revealed an overall structural similarity with slight differences in metal-ligand distances [23]. Conversion of a 2-His–1-carboxylate to a 3-His metal center was accomplished in tyrosine hydroxylase [24]. In P4H, the 3-His motif (that is, D414H P4H) did not have detectable enzymatic activity. Thus far, glutamate is the only amino acid that can replace Asp414 in P4H and maintain activity [11].

Instead of removing the carboxylate ligand, a second Asp/Glu could be added to the facial triad or the carboxylate could be relocated. His675 in aspartyl (asparaginyl) β-hydroxylase was replaced with Asp or Glu, and the enzyme retained 20 or 12% of its activity, respectively [25]. Gln or Glu could be substituted for His255 in TauD with 81 or 33% retention of its activity, respectively [19]. In P4H, however, no activity was found in the H412E or H483E variant [11]. Rearrangement of the His and Asp ligands in the P4H active site (as in the H412D/D414H and D414H/H483D variants) did not result in enzymatic activity, demonstrating that the native orientation of the 2-His–1-carboxylate facial triad is critical for P4H catalytic ability.

In conclusion, the 2-His–1-Asp facial triad in the active site of P4H is resistant to variation, including alternative iron-binding residues and the spatial positioning of the residues. Mutational analysis of the iron-binding residues within non-heme, iron(II) dioxygenases shows some flexibility at these locations, though no pattern has emerged as to which amino acids can endow function at a particular position. Although focus has been placed on the common 2-His–1-carboxylate iron-binding motif, differences among the non-heme iron(II) dioxygenases point toward the importance of residues that do not bind the iron directly. This secondary coordination sphere presumably alters the steric and electrostatic environments of the facial triad and influences the reaction mechanism. Three-dimensional structural information for human P4H, which has thus far eluded crystallization, will identify these additional residues, and could provide information that enables the design of P4H variants with transformed activity.

## Materials and Methods

### Materials

*Escherichia coli* strain Origami B (DE3) were obtained from Novagen (Madison, WI). Enzymes used for DNA manipulation were from Promega (Madison, WI) and DNA oligonucleotides for mutagenesis and sequencing were from Integrated DNA Technologies (Coralville, IA). DNA sequences were elucidated by capillary arrays on an Applied Bioscience automated sequencing instrument at the University of Wisconsin–Madison Biotechnology Center. Poly(proline) was from Sigma Chemical (St. Louis, MO). Luria–Bertani (LB) medium contained tryptone (10 g), yeast extract (5 g), and NaCl (10 g). Terrific broth (TB) medium contained tryptone (12 g), yeast extract (24 g), K_2_HPO_4_ (72 mM), KH_2_PO_4_ (17 mM), and glycerol (4 mL). All media were prepared in deionized, distilled water, and autoclaved.

### Instrumentation

UV absorbance measurements were made with a Cary 50 spectrophotometer from Varian (Palo Alto, CA). Fast protein liquid chromatography (FPLC) was performed with an AKTA system from Amersham–Pharmacia (Piscataway, NJ) and the results were analyzed with the UNICORN Control System. High-performance liquid chromatography (HPLC) was carried out with a system from Waters (Milford, MA) that was controlled with the manufacturer's Millennium32 (Version 3.20) software. Mass spectrometry was performed with a Perkin–Elmer (Wellesley, MA) Voyager MALDI–TOF mass spectrometer at the University of Wisconsin–Madison Biophysics Instrumentation Facility.

### Production and Purification of P4H Active–Site Variants

The pBK1.PDI1.P4H7 plasmid that directs the expression of the α and β subunits of human P4H served as a template for mutagenesis [26]. The QuikChange site-directed mutagenesis kit (Stratagene) was used to make point mutations in the gene encoding the α subunit. The P4H variants were produced and purified by using procedures reported previously [26]. Briefly, P4H cDNA expression in *E. coli* Origami B (DE3) cells was induced at 18°C for 18 h. Cells were harvested, lysed by sonication, and fractionated by precipitation with ammonium sulfate. P4H variants were purified by chromatography on a poly(proline)-affinity column, eluting with free poly(proline). Fractions were purified further by anion-exchange chromatography, followed by gel-filtration chromatography.

### HPLC-Based Enzyme Activity Assay

The ability of P4H to catalyze the hydroxylation or halogenation of a proline residue was monitored with an HPLC-based assay described previously [16], [26], [27]. Assays were conducted for 20 min at 30°C in 100 µL of 50 mM Tris–HCl buffer, pH 7.8, containing dithiothreitol (100 µM), catalase (100 µg/mL), ascorbate (2 mM), FeSO_4_ (50–300 µM), α-ketoglutarate (0.5–25 mM), bovine serum albumin (1.0 mg/mL), and P4H (0.09–1.5 µM). The synthetic tetrapeptide substrate (dansyl-Gly–Phe–Pro–GlyOEt) was added to initiate the reaction. A reversed-phase analytical C18 column was used to separate the substrate and product peptides. Enzymic reaction mixtures were also analyzed by mass spectrometry. To assess the effect of salt on catalysis, KF, NaCl, NaBr, NaI, KF, KCl, KBr, or KI was added to reaction mixtures to 10, 100 or 500 mM. To attempt to rescue the enzymatic activity of D414A P4H and D414G P4H, the sodium salt of azide, nitrite, nitrate, formate, or acetate was added to reaction mixtures to 100 mM.
